# Integrin signaling via FAK-Src controls cytokinetic abscission by decelerating PLK1 degradation and subsequent recruitment of CEP55 at the midbody

**DOI:** 10.18632/oncotarget.9003

**Published:** 2016-04-26

**Authors:** Siamak A. Kamranvar, Deepesh Kumar Gupta, Ying Huang, Rajesh Kumar Gupta, Staffan Johansson

**Affiliations:** ^1^ Department of Medical Biochemistry and Microbiology, Biomedical Center, Uppsala University, Uppsala, Sweden; ^2^ Department of Immunology, Genetics and Pathology, The Rudbeck Laboratory, Uppsala University, Uppsala, Sweden

**Keywords:** cytokinesis, CEP55, PLK1, integrin, FAK

## Abstract

Adhesion to extracellular matrix is required for cell cycle progression through the G1 phase and for the completion of cytokinesis in normal adherent cells. Cancer cells acquire the ability to proliferate anchorage-independently, a characteristic feature of malignantly transformed cells. However, the molecular mechanisms underlying this escape of the normal control mechanisms remain unclear. The current study aimed to identify adhesion-induced reactions regulating the cytokinesis of non-transformed human fibroblasts.

The adhesion-dependent control of cytokinesis was found to occur at a late stage close to the abscission, during which the endosomal sorting complex required for transport (ESCRT) severs the thin intercellular bridge connecting two nascent daughter cells. CEP55, a key protein involved in the abscission process, was localized at the midbody in both adherent and non-adherent fibroblasts, but it was unable to efficiently recruit ALIX, TSG101, and consequently the ESCRT-III subunit CHMP4B was missing in the non-adherent cells. PLK1, a kinase that prevents premature recruitment of CEP55 to the midbody, disappeared from this site more rapidly in the non-adherent cells. A FAK-Src signaling pathway downstream of integrin-mediated cell adhesion was found to decelerate both PLK1 degradation and CEP55 accumulation at the midbody. These data identify the regulation of PLK1 and CEP55 as steps where integrins exert control over the cytokinetic abscission.

## INTRODUCTION

Integrin-mediated cell adhesion to extracellular matrix (ECM) is required for the proliferation of normal adherent cells [1–3]. Integrins bound to ECM can activate several signaling pathways by the mechanism of integrin clustering as well as by force transmission to the associated stretch-sensitive proteins [4–7]. Cooperating signals from integrins and growth factor receptors regulate the G1-S transition of the cell cycle [8, 9] and thereby serve as a major control mechanism to avoid unregulated cell proliferation. At this checkpoint, integrin-mediated signaling is a prerequisite for the induction of cyclin D1-dependent Cdk4/6 and cyclin E-dependent Cdk2 kinase activity and the following initiation of DNA synthesis [10]. Furthermore, integrin-based signaling is implicated in the regulation of cytokinesis [3, 11–15], but the underlying molecular mechanisms remain unclear.

Cytokinesis is the final step of mitosis in which the cytoplasmic content is split between two emerging daughter cells [16]. The process of cytokinesis begins during early anaphase and proceeds sequentially in three distinct stages: cleavage furrow formation and ingression, formation and stabilization of midbody and eventually abscission [17, 18]. Contraction of an actomyosin ring causes ingression of the connected plasma membrane [19], which results in the formation of a thin intercellular bridge of densely packed microtubules oriented in antiparallel manner from the centrally located structure called midbody [20]. The midbody serves as a platform for the sequential recruitment of proteins and persists for a few hours until the abscission machinery cuts the microtubules and fuses the plasma membrane [17]. Centralspindlin, a protein complex composed of kinesin-like protein 1 (MKLP1) and MgcRacGAP, is an early component of the midbody that is involved in its linking to the plasma membrane [21]. A key step in the initiation of the abscission process is the localization of centrosomal protein 55 (CEP55) to the midbody, which occurs through binding to MKLP1 [22]. CEP55 then recruits the endosomal sorting complex required for transport I (ESCRT-I) by binding to its subunit TSG101 and to ALIX. These proteins subsequently recruit ESCRT-III subunits to the cortical rings at both sides of the midbody [23]. Several mechanisms for the final membrane fusion have been suggested, but severing of the microtubules by spastin and polymerization of ESCRT-III subunits into spiral filaments extending away from the midbody and constricting the intercellular bridge even further are evidently essential parts of the process [24–27].

Proper timing of the stepwise recruitment of midbody proteins is necessary for the successful completion of the abscission process. Polo-like kinase (PLK1) and Aurora B kinase are known to participate in this temporal regulation [28–30]. PLK1 represses ESCRT-III accumulation at the midbody by phosphorylating CEP55 at Ser436 and thereby inhibiting its interaction with MKLP1 [31, 32]. This prevents premature recruitment of CEP55 to the midbody until the late telophase when PLK1 is degraded [33].

Here we sought to identify the molecular mechanism that links cell-ECM adhesion with cytokinesis in non-transformed human fibroblasts. We report that the adhesion is required for abscission by promoting the recruitment of ALIX and TSG101 at the midbody and that integrin signaling is associated with the regulation of CEP55 through a pathway from FAK-Src to PLK1-CEP55.

## RESULTS

### Early stages of the cytokinesis process are adhesion-independent

**Figure 1:**
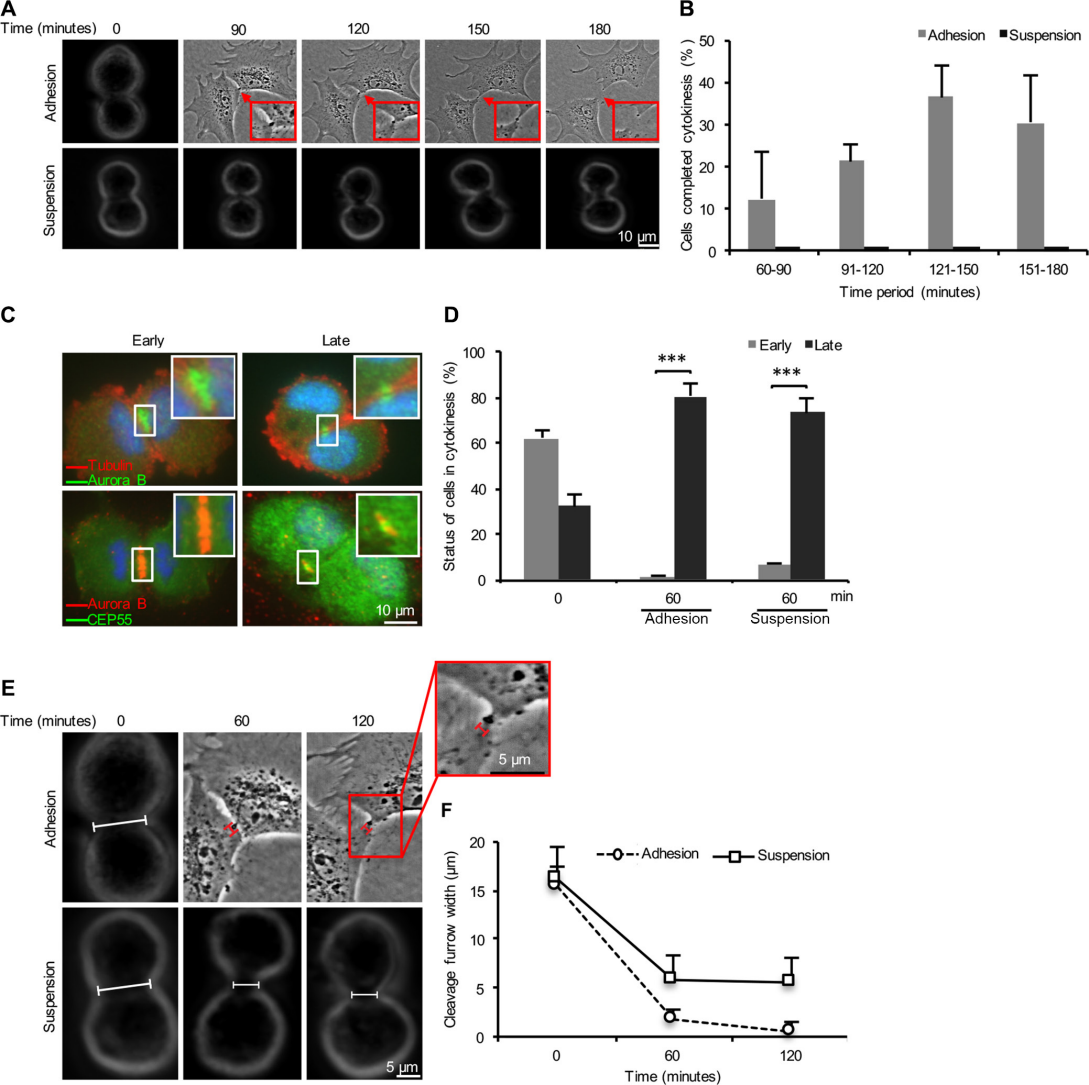
Adhesion-dependent and –independent stages of cytokinesis in non-transformed fibroblasts (**A**) Representative bright field micrographs of a single BJ fibroblast progressing through cytokinesis in the presence (adhesion) and the absence (suspension) of fibronectin contacts, respectively. (**B**) Mean % ± SD of the number of mitotic BJ fibroblasts that completed cytokinesis during the specified time periods after the formation of cleavage furrow. (**C**) Representative fluorescence images illustrating an early and a late cytokinetic stage of BJ cells. The status is based on fluorescence signal distribution of Aurora B as indicator of cleavage furrow width and the presence of CEP55 in the cleavage furrow region. Nuclei were stained with DAPI (blue). (**D**) Mean % ± SD of the number of mitotic BJ cells in the different stages of cytokinesis after the indicated incubation time periods. (**E**) Representative bright field micrographs showing the progression of the cleavage furrow ingression in the mitotic BJ cells adhering to fibronectin or kept in suspension for the indicated time periods. The bars mark the distance between the opposite membrane borders. (**F**) Mean ± SD of the cleavage furrow width. The square frames show the midbody region at higher magnification. ***P-value less than 0.001.

In order to characterize the mechanisms underlying the requirement for cell-ECM adhesion to complete cytokinesis in human non-transformed fibroblasts, we first monitored the entire process in isolated mitotic BJ cells after re-seeding in fibronectin- or Pluronic-coated plates using live-cell imaging (Figure 1A, Supplementary Videos S1 and S2). Observation of a large number of cells showed that they were able to form and ingress the cleavage furrow regardless of cell anchorage. Most adherent cells (~90%) completed the cytokinesis process within 2–3 hours after adhesion, whereas none of the non-adherent suspension cells were able to finish the process during the same period of time (Figure 1B). The formation and stabilization of midbody were then analyzed from the early phase of cytokinesis by immunostaining of selected midbody proteins. Based on the staining profile, the mitotic cells were classified as early cytokinetic cells (Aurora B-marked cleavage furrow wider than 5 μm and lacking CEP55) and late cytokinetic cells (Aurora B-marked cleavage furrow smaller than 5 μm and containing CEP55) (Figure 1C). The majority of mitotic cells (60%) were in the early stage directly after isolation by the shake-off method (0 minute time point), whereas the late stage cells were dominating (~80%) after 60 minutes incubation under either adhesive or non-adhesive conditions (Figure 1D). Progression to the late cytokinetic stage with the presence of midbody under both conditions was confirmed by the immunostaining for MgcRacGAP (Supplementary Figure S1).

### Anchorage is required for the tight constriction of cleavage furrow

Further compaction of the midbody area [22] and a subsequent secondary constriction [24, 26] are required for the membrane fusion at the intercellular bridge during abscission. The secondary constriction reduces the cleavage furrow diameter to less than 1 μm in a process where the polymerization of ESCRT-III subunits into 17 nm spiral filaments plays a critical role [24]. To assess whether cell adhesion is required for these events, we measured the width of cleavage furrows in the isolated mitotic cells after 0, 60, and 120 minutes of incubation in suspension or after seeding in fibronectin-coated plates (Figure 1E). The cleavage furrow width was reduced from 15 ± 4 μm at the 0 minute time point to approximately 5 and 2 μm, respectively, in the non-adherent and adherent cells after 60 minutes incubation. The width was further reduced to about 1 μm in the adherent cells after 120 minutes whereas it remained the same in the non-adherent cells (Figure 1F). These data indicate that the late midbody area compaction is anchorage-dependent.

### Lack of anchorage impairs the recruitment of the ESCRT machinery

**Figure 2:**
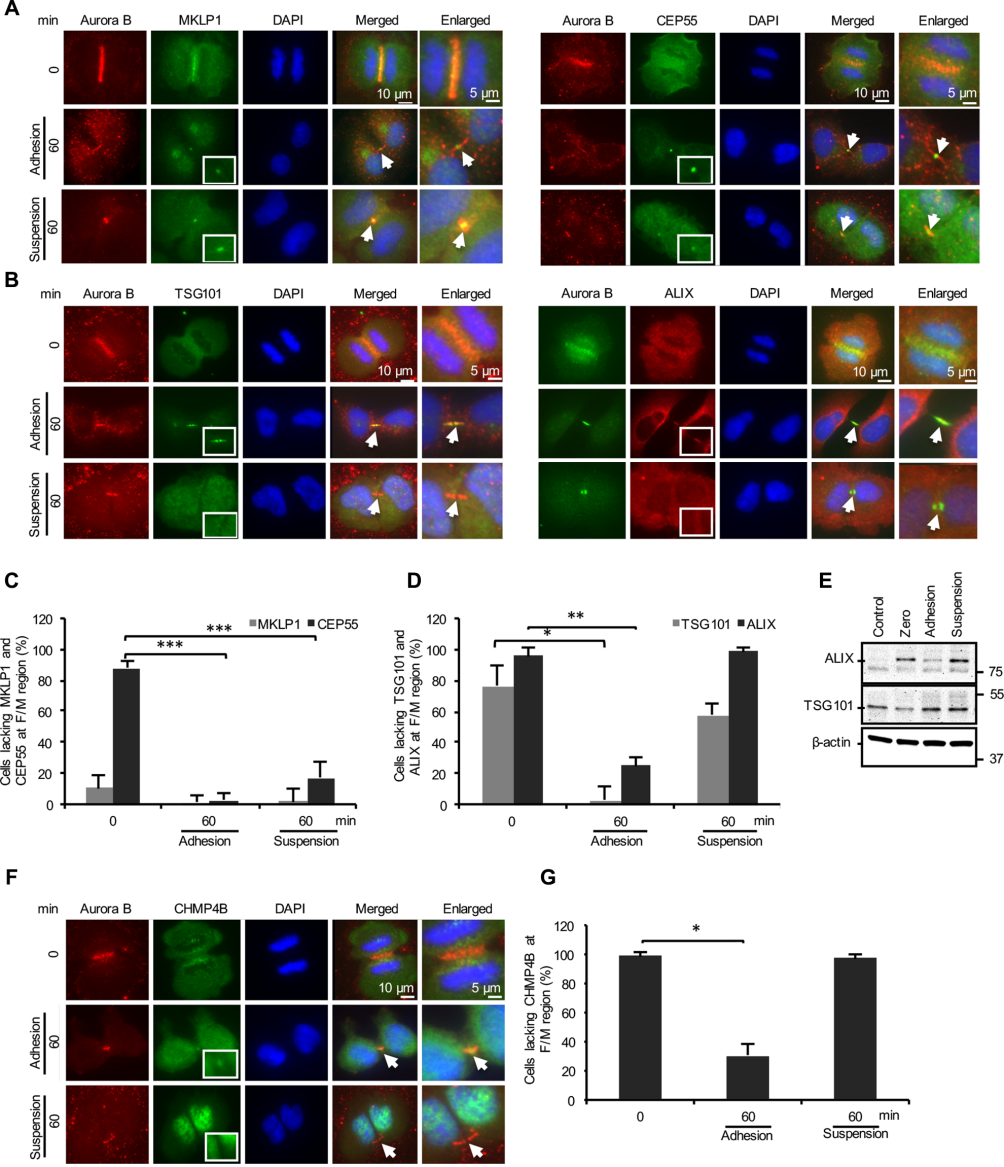
Cell adhesion is dispensable for the recruitment of the MKLP1 and CEP55 but required for the ESCRT recruitment to the midbody (**A**, **B**, **F**) Representative immunofluorescence micrographs illustrating the distribution of selected proteins in the F/M region (labeled with Aurora B) at the beginning of cytokinesis and after 60 minutes incubation of BJ cells adhering to fibronectin or kept in suspension. (A) MKLP1 (green, upper left panel) and CEP55 (green, upper right panel), (B) TSG101 (green, lower left panel) and ALIX (red, lower right panel), (F) CHMP4B (green). The square frames show the midbody area at higher magnification. Arrows indicate midbody regions in the merged pictures. Nuclei were stained with DAPI (blue). Mean % ± SD of the number of mitotic cells in cytokinesis lacking (**C**) MKLP1 and CEP55, (**D**) TSG101 and ALIX and (**G**) CHMP4B in the F/M regions. ***, **, and * represent *P*-value less than 0.001, 0.01 and 0.05, respectively. (**E**) Representative western blot of cell lysates illustrating the level of ALIX and TSG101 in exponentially proliferating cells (Control) and synchronized mitotic cells directly after isolation (Zero) and following two hours incubation under the adhesion or suspension conditions.

Next, we analyzed the localization of several midbody proteins known to be required for the abscission process in order to identify the failing cytokinetic step in the non-adherent BJ cells. MKLP1 was found in the furrow/midbody (F/M) regions of most mitotic cells at 0 and 60 minutes after incubation in suspension or on fibronectin-coated plates (Figure 2A left panel, C). As expected, CEP55 was not enriched at these sites in the initial phase of the cytokinesis but was detected at the midbody in the majority of cells after 60 minutes under both adherent and non-adherent conditions (Figure 2A right panel, C). Thus, the midbody recruitment of MKLP1 and CEP55 is anchorage-independent. In contrast, analysis of the CEP55-interacting proteins TSG101, an ESCRT-I subunit, (Figure 2B left panel) and ALIX, an ESCRT-III-binding protein, (Figure 2B right panel) revealed a significant difference between the adherent and non-adherent cells. TSG101 and ALIX were detected in the midbody of more than 90% and 70% of adherent cells, respectively, whereas the midbody staining was negative in approximately 60% of the non-adherent cells for TSG101 and in more than 90% of them for ALIX (Figure 2D). However, there was no difference (TSG101) or a moderate increase (ALIX) in the total level of these proteins in the non-adherent cells compared to the adherent cells after the 60 minutes incubation period (Figure 2E), indicating that their absence at midbodies was not due to a low expression or increased degradation in the non-adherent cells. Consistent with present models [23, 26], the absence of TSG101 and ALIX at the midbody was found to correlate with the impaired recruitment of CHMP4B, a key subunit of ESCRT-III; the number of midbodies containing CHMP4B was significantly diminished in the non-adherent cells compared with the adherent cells (Figure 2F, 2G). Similarly, spastin whose localization to the midbody is dependent on the binding to the ESCRT complex [34], was also missing in most of the non-adhering cells (Supplementary Figure S2).

### Cell anchorage is required for the abscission timing

**Figure 3:**
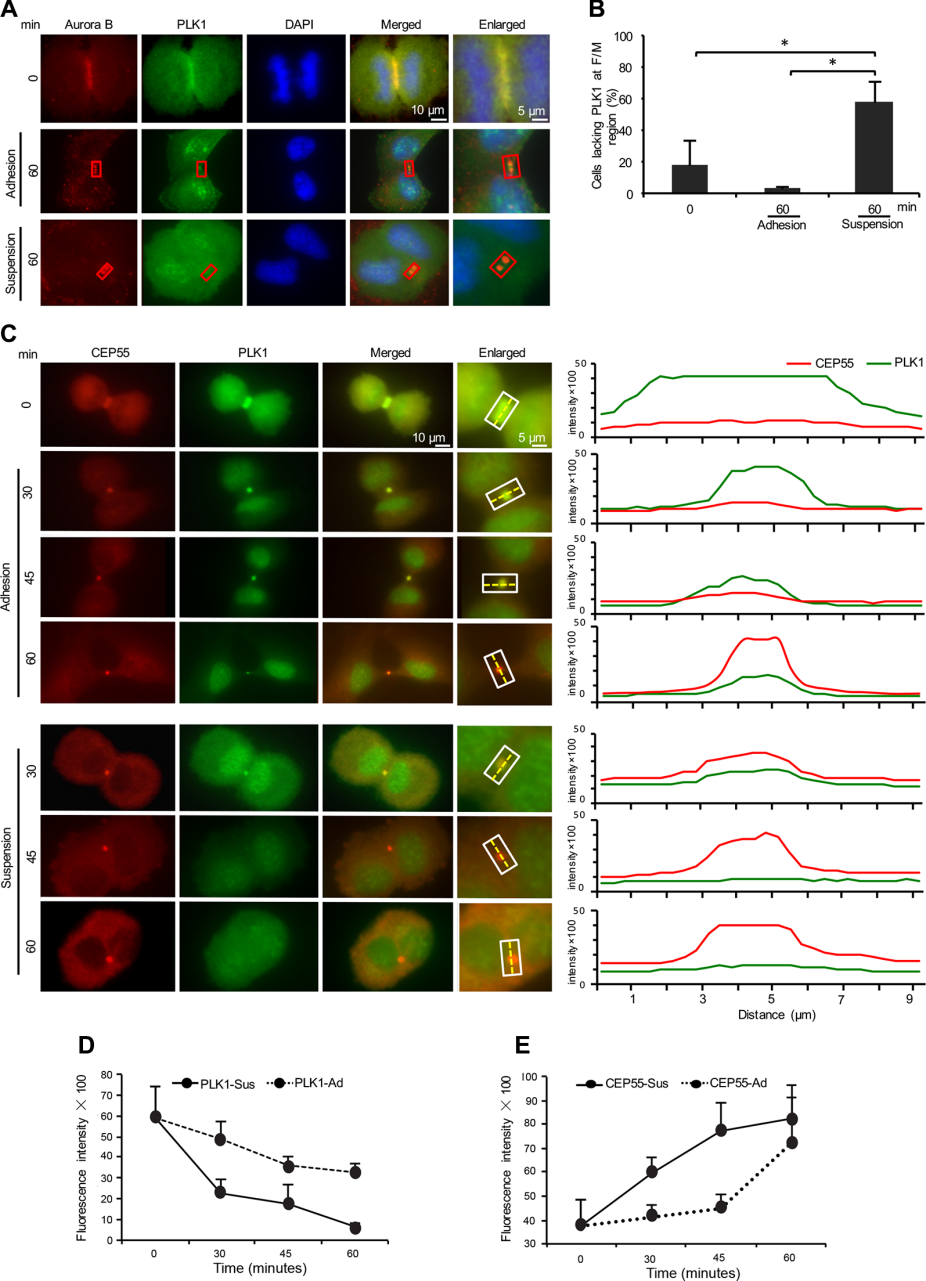
Cell adhesion is involved in the regulation of the presence of PLK1 and CEP55 at the midbody (**A**) Representative immunofluorescence images illustrating the presence of PLK1 (green) in the F/M regions stained for Aurora B (red) at the beginning of cytokinesis and after 60 minutes incubation of BJ cells adhering to fibronectin or kept in suspension. Nuclei were stained with DAPI (blue). Red squares indicate midbody areas used for the quantification of PLK1 fluorescence signal intensity. (**B**) Mean % ± SD of the number of mitotic cells in cytokinesis lacking PLK1 fluorescence signal in the F/M regions. *P-value less than 0.05. (**C**) Representative immunofluorescence micrographs showing the variation of PLK1 (green) and CEP55 (red) fluorescence signal intensity in the indicated F/M regions at the beginning of cytokinesis and in the indicated time points after the incubation of BJ cells adhering to fibronectin (upper panel) or kept in suspension (lower panel). The corresponding PLK1 and CEP55 signal intensity profiles along the hatched line in each interest area were drawn in the right panel. (**D**, **E**) Mean intensity ± SE of the PLK1 (D) and CEP55 (E) fluorescence signal intensity in the marked F/M regions 0, 30, 45 and 60 minutes after incubation of the cells adhering to fibronectin or kept in suspension (the background signal in each cell was subtracted). The fluorescence signal intensity of PLK1 and CEP55 were analyzed in 50 mitotic cells for each time point from each of three independent experiments.

One of the kinases involved in the temporal control of abscission is PLK1, which phosphorylates CEP55 and thereby prevents its early recruitment to the midbody [31, 32]. Degradation of PLK1 at the end of karyokinesis allows the localization of CEP55 to the midbody and the later completion of abscission. To study the effect of adhesion on the timing of these events, we first monitored the presence of PLK1 at the F/M regions in the adherent and non-adherent cells. PLK1 was present at the cleavage furrow region in the beginning of cytokinesis and 60 minutes later at the midbodies of most adherent cells. However, the PLK1 immunofluorescent signal was either missing or reduced close to the background level at the midbodies in a significant number of the non-adherent cells at this time point (Figure 3A, 3B). Notably, there was no apparent difference in the total level of PLK1 protein at this stage (Supplementary Figure S3).

### Lack of anchorage accelerates PLK1 degradation and the subsequent accumulation of CEP55 at the midbody

Early degradation of PLK1 results in the premature accumulation of CEP55 at the midbody and cytokinesis failure [31]. A closer analysis of the presence of PLK1 and CEP55 at midbodies of the adherent and non-adherent cells was performed by quantifying the immunofluorescence signal intensity at the midbody area for the above mentioned proteins with intervals of 15 minutes immediately after the beginning of incubation in the non-adherent or adherent conditions (Figure 3C, left panel). As the intensity profile over the midbody area from individual time points indicated (Figure 3C, right panel), the removal of PLK1 and the subsequent accumulation of CEP55 at the midbody is a gradual process which was markedly accelerated in the non-adherent cells. Thus, a significant reduction of PLK1 and subsequent accumulation of CEP55 at midbodies were seen already after 30 minutes in the non-adherent cells whereas such exchange occurred later in the adherent cells (Figure 3D, 3E).

### A FAK-Src signaling pathway controls the timing of CEP55 recruitment at the midbody

**Figure 4:**
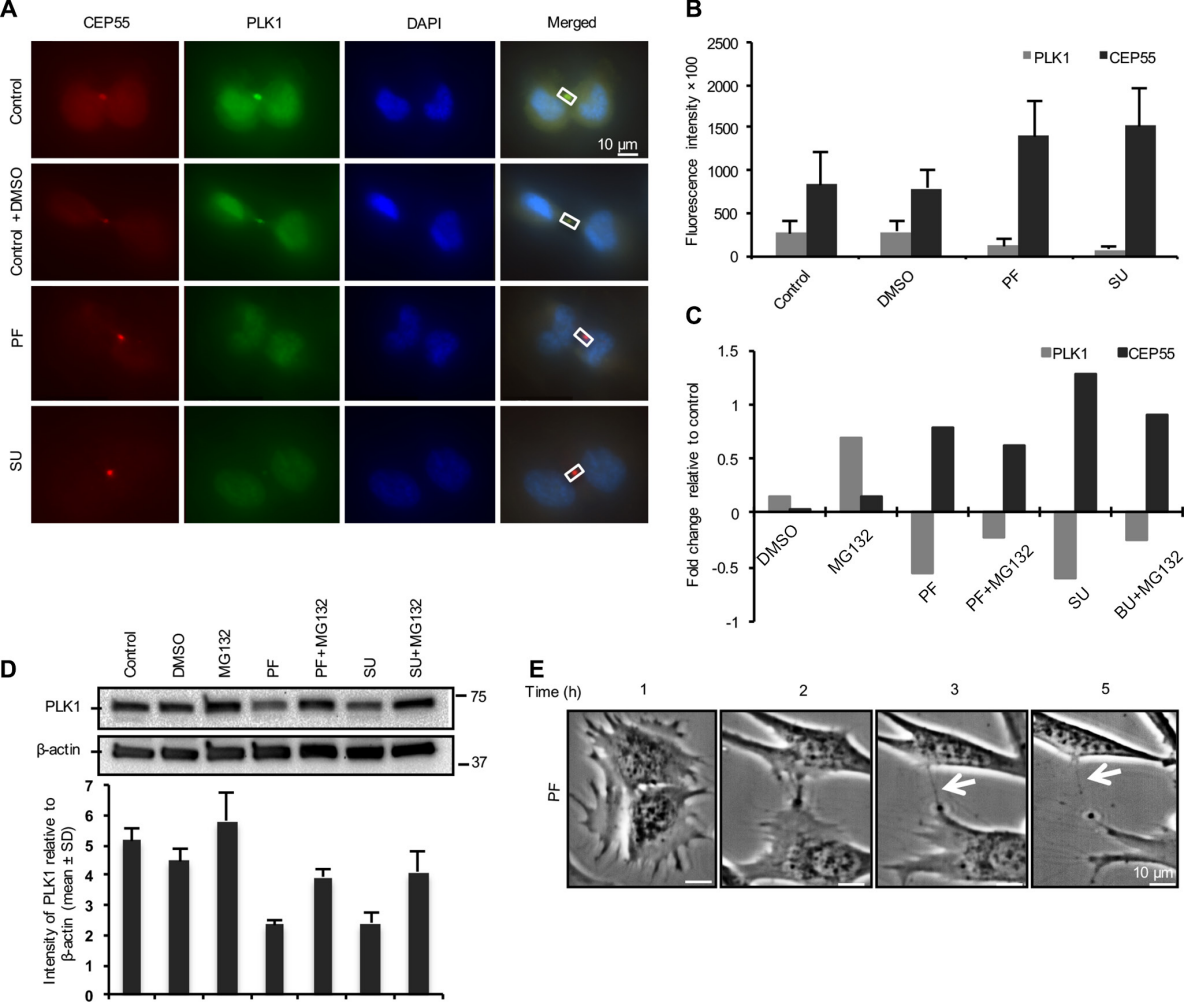
FAK-Src signaling is involved in abscission timing through PLK1 controlling the recruitment of CEP55 (**A**) Representative immunofluorescence images showing the PLK1 (green) and CEP55 (red) signals at the midbody of mitotic BJ cells in cytokinesis replated on fibronectin and treated with DMSO, FAK inhibitor (PF) and Src inhibitor (SU) for 1 hour. (**B**) Quantification of PLK1 and CEP55 signals at the midbody of cells shown as mean ± SD. (**C**) The variation in the PLK1 and CEP55 fluorescence signal intensities presented as fold change relative to control values after the treatment of the cells with the indicated inhibitors. (**D**) Representative western blot and quantification analysis (mean ± SD; *n* = 2) showing the expression level of PLK1 in the mitotic cells treated as described above. (**E**) Representative bright field micrograph illustrating the progression of cytokinesis during the indicated time period in a single BJ fibroblast treated with FAK inhibitor (PF). The arrows mark the uncut intercellular bridge between two daughter cells.

In attempts to identify integrin-mediated signals involved in the temporal regulation of PLK1 and CEP55, we used inhibitors of Src, FAK, PI3K, PLC3, MEK, ROCK and microtubule-dependent vesicle transport to block the selected main signaling pathways. Mitotic cells were treated with the inhibitors 15 minutes after replating on fibronectin-coated coverslips, and 45 minutes later the cells were fixed and immunostained for PLK1 and CEP55. Quantification of the fluorescent signals revealed that the inhibition of either FAK or Src in the adherent fibroblasts resulted in reduced PLK1 and enhanced CEP55 signals at the midbodies, similar as in the non-adherent cells (Figure 4A, 4B). The other tested inhibitors did not have a comparable effect (Supplementary Figure S4).

Inhibition of proteasome-dependent protein degradation with MG132 treatment diminished the effect of FAK and Src inhibitors on the temporal regulation of PLK1 at the midbody (Figure 4C), indicating that a FAK-Src signaling pathway may control the timing of the PLK1 degradation process in the BJ cells. This conclusion was supported by western blot analysis (Figure 4D) of lysates from cytokinetic cells treated as above. However, for unknown reasons, the MG132 treatment only marginally reduced the level of CEP55 at the midbody (Figure 4C).

Finally, we asked if the inhibition of FAK-Src signaling could block the cytokinetic abscission. The mitotic cells treated with FAK inhibitor were monitored by live imaging for 5 hours during incubation on fibronectin-coated plates (Figure 4E, Supplementary Video S3). The mitotic cells could proceed in the cytokinesis and form a narrow intercellular bridge, but abscission did not occur even when cell migration stretched the thin bridge, indicating that FAK-Src signaling is required for the final cleavage.

## DISCUSSION

One of the characteristics of cancer cells is the capability to proliferate independently of integerin-mediated ECM adhesion, which normally controls cell cycle progression through the G1 phase and the cytokinesis. Suppression of the G1/S checkpoint, e.g. by inactivating mutations of pRb and p53 or by viral proteins, is required for the tumorigenic transformation of cells. Thus, the adhesion-dependence for cytokinesis has been suggested to be a protective mechanism against metastasis of cells with suppressed G1/S checkpoint by preventing proliferation of detached or miss-located cells [35]. However, this potential control mechanism is circumvented in malignant tumor cells [36] by poorly understood mechanisms. Furthermore, it may be a double-edged sword that also can promote tumor progression. Failure to complete cytokinesis, e.g. by interference with integrin function, results in the formation of bi-nucleated cells [11, 13, 15, 37, 38], which will cause chromosomal instability in the subsequent rounds of the cell cycle [39, 40]. Thus, better understanding of the molecular mechanisms underlying the normal cell adhesion control of cytokinesis and the anchorage-independent cytokinesis of tumor cells is important for the further improvement of cancer treatment.

Much of the present knowledge on the regulation of the cytokinesis has been obtained from studies in HeLa cells, but previous studies indicate that the regulation of the process contains cell type-specific features that differ between epithelial cells, fibroblasts and mesenchymal stem cells [12, 41]. To identify which phase(s) of cytokinesis is affected by integrin-mediated adhesion in non-transformed human fibroblasts in an unprejudiced manner, we analyzed the entire process in details and found that it was halted downstream of CEP55. It has been recently shown that the timing of CEP55 recruitment to midbody for unknown reasons is critical for the functionality of this protein in the promotion of successful abscission [31]. PLK1-mediated phosphorylation of CEP55 at Ser436 prevents premature arrival of CEP55 to the midbody until the late anaphase, at which point PLK1 is degraded. Inhibition of PLK1 or expression of CEP55 mutated at the PLK1 phosphorylation site (S436A) results in untimely accumulation of CEP55 at the midbody and abscission failure [31, 32]. The quantification of the PLK1 immunofluorescence signal at the midbody of non-adherent cells indicated either absence or a significant reduction of this protein compared to the adherent control cells (Figure 3). This suggests that adhesion may contribute to the proper temporal recruitment of CEP55 by regulating PLK1 localization at the midbody. According to Bastos et al., rapid recruitment of CEP55 due to early degradation of PLK1 results in the accumulation of “immature” CEP55, which is not able to recruit ESCRT components to complete abscission [31]. The same scenario may be applied to the non-adherent BJ cells through an un-coupling between integrin signaling, CEP55 regulation and abscission. These studies underscore the need to identify what the proposed CEP55 immaturity consists of.

Among the tested chemical inhibitors against several signaling pathways which may be involved in this process upstream of PLK1/CEP55, FAK and Src inhibitors induced a significant reduction of PLK1 and accumulation of CEP55 at the midbody of adherent cells (Figure 4A, 4B). FAK is known to recruit Src family kinases to focal adhesions and thereby activate several signaling pathways, which are crucial for cell migration, survival and proliferation [42, 43]. The effect of FAK and Src inhibitors on PLK1 was reduced by treatment with the proteasome inhibitor MG132 (Figure 4C, 4D), supporting the conclusion that integrin-activated FAK/Src may delay PLK1 degradation. Live cell imaging of the adherent cells treated with the FAK inhibitor revealed the abscission failure even when the cell migration exerted strong mechanical force on the intercellular bridge, illustrating that FAK-dependent signals rather than mere pulling force [44, 45] is needed to complete abscission. However, evidence was recently presented that mechano-signals from traction force also contribute to the abscission process in dermal fibroblasts [41]. That study showed that more cells (23%) were unable to complete cytokinesis on a soft matrix than on a stiff matrix (4%) although in both cases the cells were adhering via integrins to fibronectin. Interestingly, a mesenchymal stem cell line completed cytokinesis equally well on the stiff and soft matrices [41]. Integrins can generate different signals by at least two distinct mechanisms, i.e. by ligand-induced clustering and by mediating force to stretch-sensitive proteins [7], and the mesenchymal stem cell data suggest that signals triggered by integrin ligand binding is sufficient to promote cytokinesis. The question then arises why the dermal fibroblasts needed both types of integrin stimuli? Our study shows that FAK activity was needed for the abscission in BJ fibroblasts adhering to the fibronectin-coated surface. FAK is known to be activated by integrin clustering [5], yet its activity in BJ fibroblasts was previously found to be enhanced by traction force acting on adhesion sites [7]. Since the activation mechanism of FAK does not require a force-induced conformational change [46], the stimulatory effect of traction force in some cell types (including dermal and BJ fibroblasts) may be due to secondary events affecting the phosphorylation status of the protein (e.g. inhibition of phosphatase activity).

Cytokinesis in several epithelial cell lines was recently reported to require integrin-induced activation of ERK, in contrast to human fibroblasts [12]. In agreement with that study, we found that the blocking of the ERK pathway by the inhibition of MEK did not prevent cytokinesis of adherent BJ cells (not shown) and had no major effect on the timing of CEP55 recruitment to midbodies (Supplementary Figure S4). Similar results were obtained with inhibitors targeting PI3K, PLC3 or ROCK. Inhibition of ROCK was previously reported to interfere with cytokinesis [3], which may seem to oppose our results. However, the inhibitor (Y-27632) given to the cells in pre-metaphase in the study by Tullberg et al. presumably affected the cells already at the furrow ingression stage, while we added the inhibitor later and specifically analyzed CEP55 in the cells which had formed the midbody as detected by Aurora B staining.

In conclusion, the step in the abscission process that is blocked in detached normal fibroblast cells was in this report pinpointed to the interactions CEP55–ALIX and CEP55–TSG101. It also identified aberrant temporal control of PLK1 and CEP55 at the midbody as a plausible cause for the blocked step. Additionally, FAK and Src were found to be necessary components in the integrin-mediated signaling pathway regulating PLK1, CEP55 and abscission. Further investigations are required to fill in the gaps in our understanding of this fundamental process that is deregulated in tumor cells. In particular, this study highlights the need to clarify the mechanisms regulating PLK1 degradation and CEP55 interactions.

## MATERIALS AND METHODS

### Cell lines and culturing of mitotic cells

The human non-transformed fibroblast cells (hTERT-immortalized BJ cells) were cultured in Dulbecco's modified Eagle medium (DMEM, Gibco, Life technologies, UK) supplemented with 10% fetal bovine serum (FBS, FB-1090-500, Werner Saveen, Biological Industries, Beit-Haemek Ltd, Israel), 100 U/ml penicillin and 0.1 mg/ml streptomycin (complete medium). The cells were kept at 37°C in a humidified atmosphere containing 5% CO_2_.

Mitotic cells were collected by the shake-off method [47] in which exponentially growing cells were washed once with pre-warmed PBS followed by incubation in the complete medium for approximately 3 hours after which the loosely attached mitotic cells were detached by tapping the culture flasks. The floating mitotic cells were then collected by centrifugation and resuspended in the fresh complete medium for the culturing in plates coated with either Pluronic (10 mg/ml, F108 NF Prill Poloxamer 338, D-BASF, Germany) for the adhesion-independent condition (suspension) or fibronectin (40 μg/ml) |for the adhesion-dependent condition (anchorage). Where indicated, the cells were treated with the following inhibitors for 1 hour prior to the fixation: FAK/PYK inhibitor PF-562271 (5 μM, 2BScientific, UK), Src family inhibitor SU-6656 (10 μM, Sigma Aldrich, Saint Louis, USA), MEK inhibitor PD98059 (50 μM, Calbiochem, San Diego, USA), proteasome inhibitor MG132 (100 μM, Calbiochem, San Diego, USA), Nocodazole (50 ng/ml, Sigma Aldrich), phospholipase 3 inhibitor, PLC3 sc-3574 (5 μM, Santa Cruz, USA), PI3 kinase inhibitor, LY294002 (25 μM, Cell Signaling Technology, USA) and ROCK inhibitor, Y-27632 (20 μM, Tocris Bioscience, Bristol UK).

### Live-cell imaging

Live cell imaging was performed using an inverted microscope (Nikon-Eclipse Ti-U, Japan) equipped with a CCD camera (Andor's multi pixel sCMOS camera, Oxford Instruments) and a cell culture chamber having constant supply of humidified 5% CO_2_ and temperature control. Adherent cells were monitored in the fibronectin- coated culture plates whereas non-adherent cells were monitored in ultra-low attachment 6-well cell culture plates (Corning incorporated NY 14831, USA) and in the complete medium containing 0.005% agarose (Ultra-Pure, Invitrogen, USA). The images were acquired using 40 × magnification objective and phase contrast filter of the time-lapse microscope in 2-minutes time intervals for 5 hours.

### Cell synchronization in G2/M phase

To synchronize a large number of mitotic cells for western blotting, a combined method was used. The cells were first subjected to 24 hours of serum starvation (0.1% serum) followed by incubation in the complete medium containing 5 mM thymidine (Sigma Aldrich) for 24 hours. After two hours release from the thymidine block by three times washing with PBS, the cells were then treated with Nocodazole (50 ng/ml) for 14 hours. The synchronized mitotic cells were then harvested using shake-off method as described above. To release mitotic cells from the Nocodazole block, the cells were washed twice with PBS and once with the complete medium. The mitotic cells were then incubated for 2 hours with the complete medium in order to allow them to enter into the phase of cytokinesis prior to lysing.

### Immunostaining and quantification of fluorescent signal

For the adhesion-dependent condition, the mitotic cells were cultured on fibronectin-coated coverslips whereas they were cultured in Pluronic-coated plates for the adhesion-independent condition and thereafter deposited on glass slides by cytospin centrifugation. Subsequently, the cells were fixed by cold methanol at −20°C for 20 minutes and then washed twice in PBS for 5 minutes. After incubation in PBS containing 1% BSA (Fraction V Roche Diagnostic, Germany) and 0.1% Tween20 (Merck, Germany) (blocking buffer), the slides were incubated overnight at 4°C with primary antibodies diluted 1:50 in blocking buffer. Antibodies directed against the following proteins were used: Aurora B (ab-2254, rabbit polyclonal and ab-3609 mouse monoclonal, Abcam, Cambridge, UK), CEP55 (sc-134622, rabbit polyclonal and sc-37405, mouse monoclonal, Santa Cruz, California, USA), PLK1 (GTX-104302, rabbit polyclonal, GENTEX, Zeeland, USA), ALIX (3A9, mouse monoclonal, Thermo Fisher scientific, Waltham, USA), TSG101 (GTX-118763, rabbit polyclonal, GENTEX,), α-tubulin (T6199, mouse monoclonal, Sigma, Saint Louis, USA), MKLP1 (sc-22793, rabbit polyclonal, Santa Cruz), CHMP4B (sc-82556, rabbit polyclonal, Santa Cruz), Spastin Sp 3G11/1 (sc-53443, mouse monoclonal, Santa Cruz). The slides were then washed with PBS and incubated for 1 hour with secondary antibody (diluted 1:500 in blocking buffer, Alexa Fluor 488-conjugated goat anti-rabbit and Alexa Fluor 594-conjugated goat anti-mouse, Invitrogen, Carlsbad, USA), washed with PBS and mounted with medium containing DAPI (4,6-diamidino-2-phenylindole, Invitrogen). Digital images of the cells were captured using a Nikon fluorescence microscope (Nikon Eclipse 90i, Japan) equipped with a CCD camera (DS-Qi1 Monochromatic Digital Camera). The digital images were analyzed for the presence or absence of immunostained proteins at the midbody and scored using Adobe Photoshop^©^ (Adobe Photoshop CS6, Adobe system Inc. San Jose, CA, USA) and ImageJ (http://rsb.info.nih.gov) software. After subtraction of the background signal for each cell, the fluorescence intensity and signal intensity profile of each identified midbody area was measured using the NIS software (NIS Elements, Nikon). For all the experiments 25–50 randomly selected cells per condition and for each time point were analyzed from each of three independent experiments.

### Western blotting

Total cell lysates were prepared in lithium dodecyl sulfate (LDS, CA 92008, Novex, Life technologies) sample buffer, fractionated in precast 4–12% SDS-PAGE gradient gels (Biorad, Mini-Protean-TGX, USA) and transferred to nitrocellulose membrane (Thermo Scientific, 3747 N, Meridian Rd. Rockford, USA). The blots were probed with primary antibodies to Aurora B (1:1000, ab-2254, Abcam), CEP55 (1:1000, sc-134622, Santa Cruz), PLK1 (1:1000, GTX 104302, GENTEX), ALIX (1:500, sc-53538, Santa Cruz), TSG101 (1:250, GTX 118763, GENTEX) and β-actin (1:5000, ab 6276-100, Abcam) followed by the appropriate HRP-conjugated secondary antibody (HRP-conjugated donkey anti-rabbit, NA9340V and HRP-conjugated sheep anti-mouse, NA9310V, GE Healthcare) and developed by the enhanced chemiluminescence method (Amersham ECL, GE Healthcare Life Sciences) and analyzed using Image Lab software (Version4, Bio-Rad Laboratories).

### Statistical analysis

The statistical analysis was performed using student's *t*-test. *P*-values < 0.05 were considered as significant.

## SUPPLEMENTARY FIGURES AND TABLES








