# Supplementary material for: Integrin signaling via FAK-Src controls cytokinetic abscission by decelerating PLK1 degradation and subsequent recruitment of CEP55 at the midbody
Source: Oncotarget. 2016 Apr 26;7(21):30820–30. doi: 10.18632/oncotarget.9003 (PMC5058720; doi:10.18632/oncotarget.9003)
Supplement: Supplementary file 1 [file oncotarget-07-30820-s001.pdf]

Integrin signaling via FAK-Src controls cytokinetic abscission by decelerating PLK1 degradation and subsequent recruitment of CEP55 at the midbody

Supplementary Materials

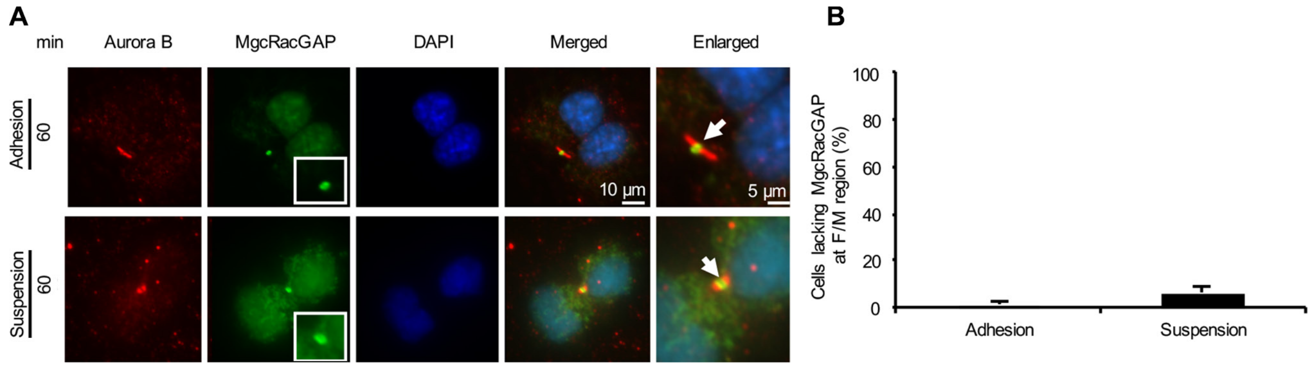

Supplementary Figure S1: Cell adhesion is dispensable for the formation of midbody. (**A**) Representative immunofluorescence images illustrating the presence of MgcRacGAP (green) in the midbody region marked with Aurora B (red), 60 minutes after incubation of BJ cells adhering to fibronectin or kept in suspension. Nuclei were stained with DAPI (blue). The square frames show the midbody region at higher magnification. (**B**) Mean % ± SD of the number of mitotic cells in cytokinesis lacking MgcRacGAP signal (green) at the midbody region.

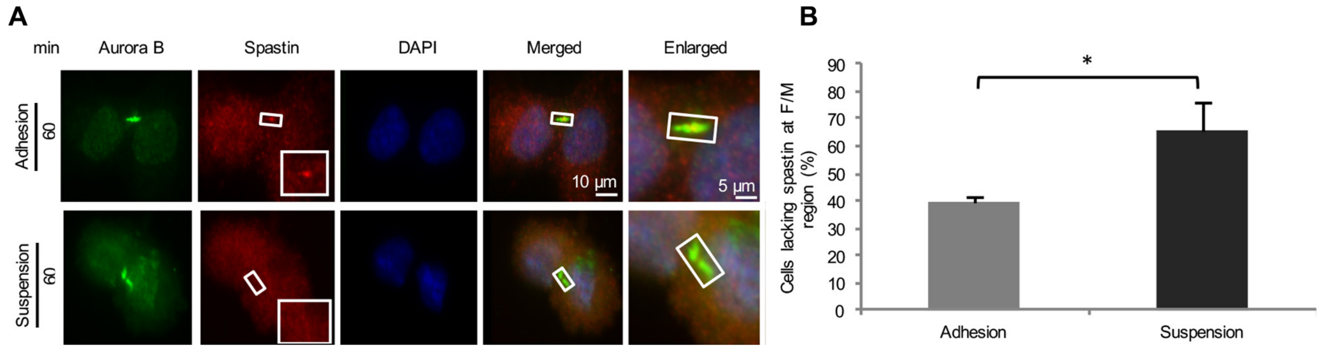

Supplementary Figure S2: Cell adhesion is required for the spastin recruitment to the midbody. (**A**) Representative immunofluorescence micrographs showing the presence of spastin (red) in the midbody region stained for Aurora B (green), 60 minutes after incubation of BJ cells adhering to fibronectin or kept in suspension. Nuclei were stained with DAPI (blue). White squares indicate midbody areas used for the quantification of spastin fluorescence signal intensity. (**B**) Mean % ± SD of the number of mitotic cells in cytokinesis lacking spastin signal in the midbody region with higher intensity than the cytoplasmic background level. *P-value less than 0.05.

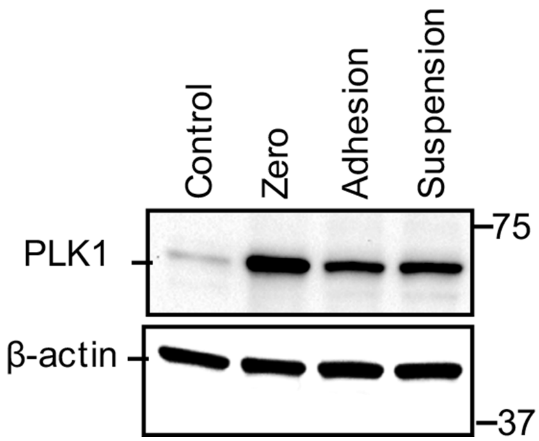

Supplementary Figure S3: Comparison of PLK1 expression level in the adherent and suspension BJ fibroblast cells. Representative western blot of cell lysates illustrating the level of PLK1 in exponentially proliferating cells (Control) and synchronized mitotic cells directly after isolation (Zero) and following two hours incubation under the adhesion or suspension conditions.

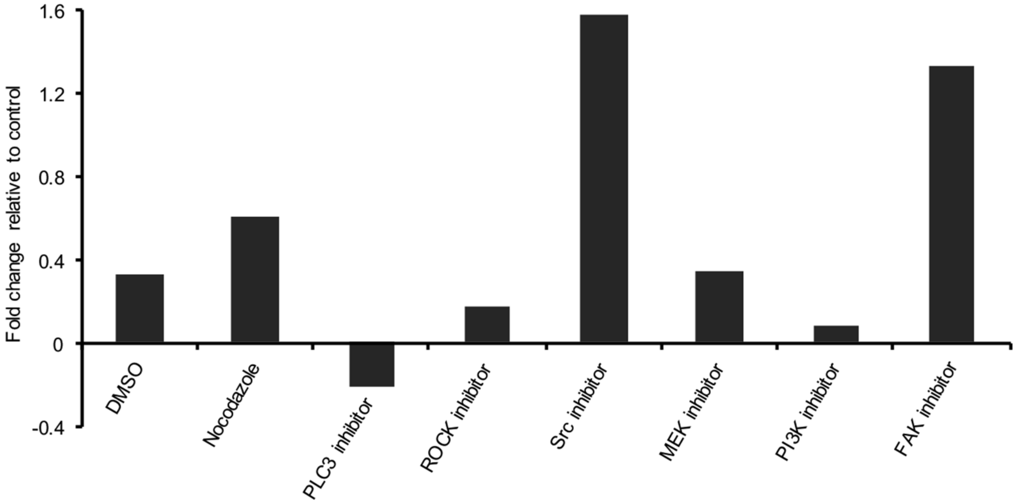

Supplementary Figure S4: The effect of selected inhibitors on the recruitment of CEP55 to the midbody. Mitotic BJ cells were isolated and replated on fibronectin and after 15 minutes, they were treated with the indicated inhibitors for 1 hour. The signal from immunofluorescent staining of CEP55 was quantified in the cells with midbodies marked with Aurora B antibodies. The variation in the CEP55 fluorescence signal intensity is presented as fold change relative to control values.

Supplementary Video S1: This video shows the process of cytokinesis from the cleavage furrow formation to the abscission in a BJ fibroblast cell adhering to fibronectin.

Supplementary Video S2: This video illustrates the process of cytokinesis from the cleavage furrow formation in a BJ fibroblast cell kept in suspension.

Supplementary Video S3: This video shows the process of cytokinesis in the adherent BJ cells treated with FAK inhibitor (PF). The arrow marks the stretched narrow intercellular bridge that remained uncut between two daughter cells.
